# Optimizing production of serially diluted compounds and distribution to multiple targets

**DOI:** 10.1155/S1463924601000220

**Published:** 2001

**Authors:** Cole O. Harris, Stephanie L. Schweiker

## Abstract

The need for a multiple-target compound selectivity programme led to the establishment of a single robotic system that produces a compound's serial dilution and its distribution to multiple replicate assay plates. A Genesis RSP 150 integrated into a Zymate Laboratory Automation System XP produced the serial dilutions, and the subsequent replicate assay plates were produced quickly and accurately by an efficient use of the carousels and rapid plate. Currently, this process allows for the production of over 200 serial dilution assay plates in a workday.


Journal of Automated Methods & Management in Chemistry, Vol. 23, No. 6 (November–December 2001) pp. 179–182

### Optimizing production of serially diluted compounds and distribution to multiple targets†

**Cole O. Harris and Stephanie L. Schweiker**
*GlaxoWellcome Research and Development*

*The need for a multiple-target compound selectivity programme led to the establishment of a single robotic system that produces a compound's serial dilution and its distribution to multiple replicate assay plates. A Genesis RSP 150 integrated into a Zymate Laboratory Automation System XP produced the serial dilutions, and the subsequent replicate assay plates were produced quickly and accurately by an efficient use of the carousels and rapid plate. Currently, this process allows for the production of over 200 serial dilution assay plates in a workday.*

### Introduction

With the level of high-throughput screening (HTS) achievable today, there is an ever-increasing demand for the production of serially diluted compound assay plates for secondary screening, structure-activity relationship (SAR) development and target specficity. Because there is not a machine on the market capable of making these dilutions in DMSO at volumes required for assays, these dilutions must be transferred at low volume to an assay plate.

A successful approach to a standard application for producing serial dilutions using a TECAN liquid handler was combined with a Zymark track system to accomplish the entirety of the task on a single machine.

### Concept

A master plate is retrieved from the first carousel and placed on the deck of the Tecan for serial dilution. The RapidPlate 96/384 (Zymark, MA, USA) was loaded with tips and the previous serially diluted plate. The subsequent copy plates for assays were then retrieved from the carousels and copies of the dilution made as the next serial dilution was proceeding. Each plate was sealed and returned to its original location. As each plate was retrieved and/or returned, the RapidPlate was executing a transfer function.

### *System*

Serial dilutions are commonly peformed in DMSO to avoid the compound precipitation. Assay stringencies were met by transferring 1μl samples from this dilution plate for the production of multiple-copy assay plates. This approach accommodates a range of low-volume assays with a standard volume of compound while maintaining a low percentage of DMSO. These identical copy plates were then distributed to assays within a systems-based research area for the purpose of specificity screening.

An important component of this automated process is receiving a compound as a liquid sample from a central distribution group as a standard format of compounds on a master plate. (From earlier work, it was demonstrated that compound distribution of liquid samples doen ot affect quality of compound stability.) The format that simplified and most benefited the automated process was the 'column-daughter' arrangement (figure 1). These plates were designed with consideration for robot handling and to facilitate programming. For ease of plate handling and deck efficiency, the 'column-daughter' format eliminated the need for a master plate containing 80 compounds to be distributed to plates that would be subsequently diluted. The easiest step to automate is the step that you do not do.

Another consideration in simplifying and increasing the efficiency of the process is recognizing that on a track or rail system, the gripper/arm is the bottleneck. By minimizing the number of moves the arm travels while unoccupied, the efficiency of the system increases. This consideration led to the coordination of three carousels and a single rapid plate to produce the copy plates rapidly.

### *Carousels*

The goal was to retrieve the plates from the carousels in an order that eliminates the carousel start-up/shut-down times from the plate acquisition process and this could be achieved by making the next plate in the process always come from a different carousel. With this arrangement of carousels, such that the next plate to be put into process would never interefere with the last plate returned, the time lost in carousels processing plates was minimized, i.e. carousel 1: returned plate, most recent copy, carousel 2: plate active in RapidPlate, copy being made, carousel 3: next plate to enter process, 'start-up' does not interfere with retrieval of next plate (figures 2 and 3).

### *Tecan Genesis RSP 150*

Serial dilutions using standard tips were easy to validate with dyes in DMSO. However, the accuracy of the serial dilutions became compromized with the introduction of

† This paper was initially presented at the ISLAR 2000 Conference and is reproduced here by kind permission of Zymark Corporation.

*Journal of Automated Methods & Management in Chemistry* ISSN 1463–9246 print/ISSN 1464–5068 online © 2001 ISLAR
http://www.tandf.co.uk/journals
DOI: 10.1080/14639240110092549

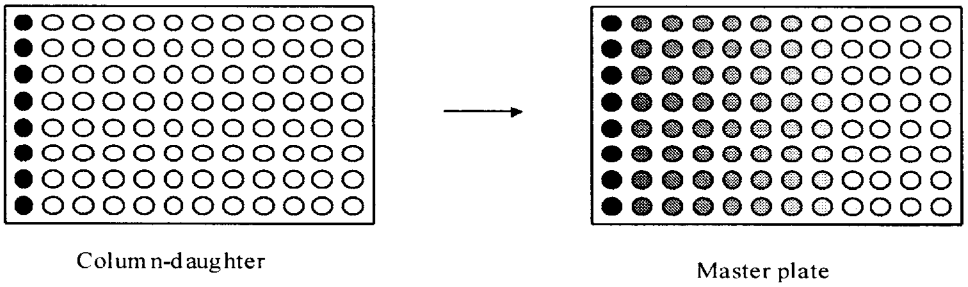

| | | | | | | | | | | | |
|---|---|---|---|---|---|---|---|---|---|---|---|
| 3.349 | 3.285 | 3.197 | 1.629 | 0.563 | 0.19 | 0.087 | 0.022 | 0.022 | 0.021 | 0.006 | 0.024 |
| 3.355 | 3.32 | 1.549 | 0.54 | 0.179 | 0.081 | 0.019 | 0.005 | 0.021 | 0.022 | 0.104 | 0.028 |
| 3.374 | 1.518 | 0.524 | 0.186 | 0.083 | 0.019 | 0.007 | 0.003 | 0.025 | 0.026 | 0.023 | 0.024 |
| 1.697 | 0.552 | 0.193 | 0.066 | 0.022 | 0.006 | 0.002 | 0 | 0.025 | 0.023 | 0.008 | 0.023 |
| 0.575 | 0.182 | 0.067 | 0.027 | 0.006 | 0.001 | 0 | -0.001 | 0.028 | 0.024 | 0.024 | 0.024 |
| 0.2 | 0.064 | 0.035 | 0.007 | 0.001 | -0.001 | -0.001 | -0.001 | 0.027 | 0.025 | 0.02 | 0.026 |
| 0.075 | 0.023 | 0.009 | 0.001 | 0 | -0.001 | 0 | -0.001 | 0.029 | 0.027 | 0.027 | 0.025 |
| 0.026 | 0.009 | 0.004 | 0.002 | 0 | -0.001 | 0.002 | -0.001 | 0.03 | 0.027 | 0.006 | 0.028 |

*Figure 1. 'Column-daughter' plate contains chemistry in DMSO in column 1 only. DMSO is added to columns 2–12 as the diluent and control for the serial dilution. The chemistry in column 1 is then diluted across the plate through column 11 to produce the serial dilution master plate for distribution to assay plates.*

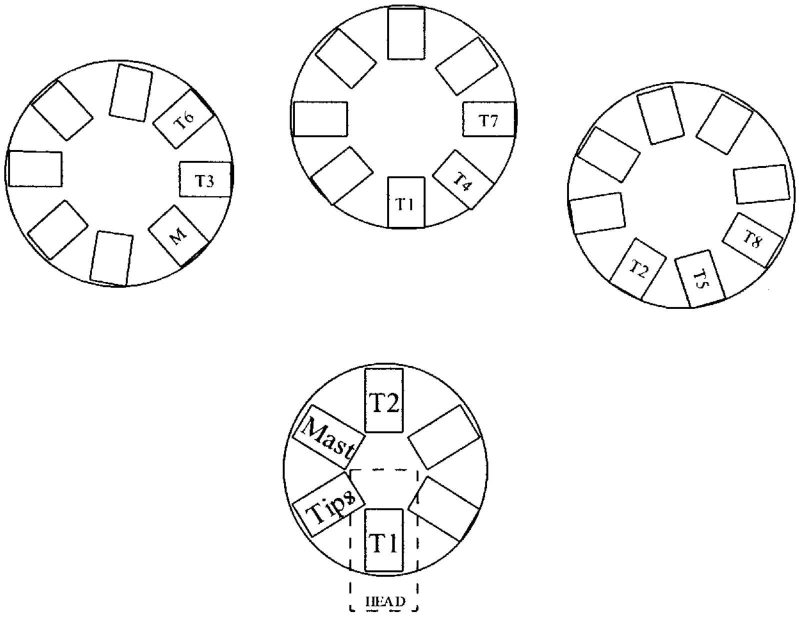

*Figure 2. Racks that contain each defined assay plate are staggered in the carousels to allow for plate processing to occur without delay from the ejecting or parking of a plate. This is important for the efficiency of the system because of the constant interaction between the carousels and the RapidPlate (figure 3).*

real compounds because of carry-over issues specific to compound classes. While using standard tips, this process required an escalating number of washes to compensate for carry-over, resulting in an increasing volume of liquid waste and a significant amount of additional washing time. A standard serial dilution application for any compound class was needed to automate the task successfully.

Through extensive work for the production of compound doses with Tecans, the most successful and reproducible approach incorporates 250 µl syringes and disposable polypropylene tips. Tips (200 µl) are used for convenience and accuracy to fill the plate with the diluent DMSO. Tips (50 µl) are used for accuracy in the serial dilution of the compounds, diluting across the plate for 11 concentrations and leaving column 12 to contain only DMSO for assay controls. The tips are not changed between individual concentrations but rather at the end of each serial dilution.

A Tecan Genesis 150 with Gemini (Tecan, Ag) as the controlling software for the serial dilution program allows for tighter control of the Tecan instrument and yields a more accurate result. Goodness of fit data ($r^2$ from least-squares curve-fitting) with real chemistry demonstrates the accuracy of this approach (table 1). While the previously diluted plate is being distributed to the copy plates, the current serial dilution is proceeding.

*RapidPlate*

Employing a rapid plate structure that allows the arm to place a plate on the liquid handler and return with a

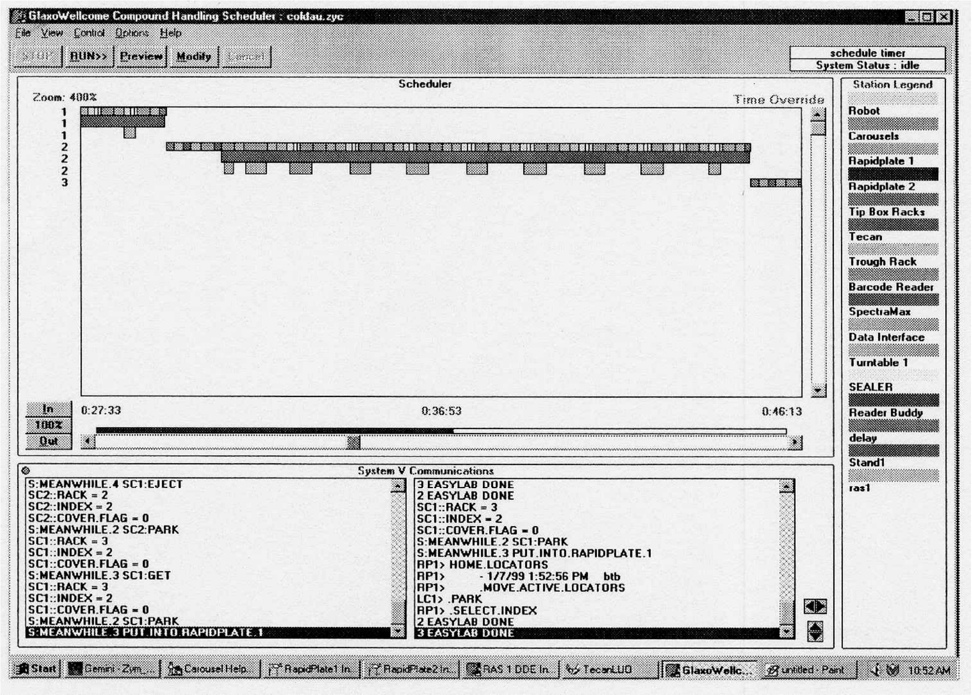

(A)

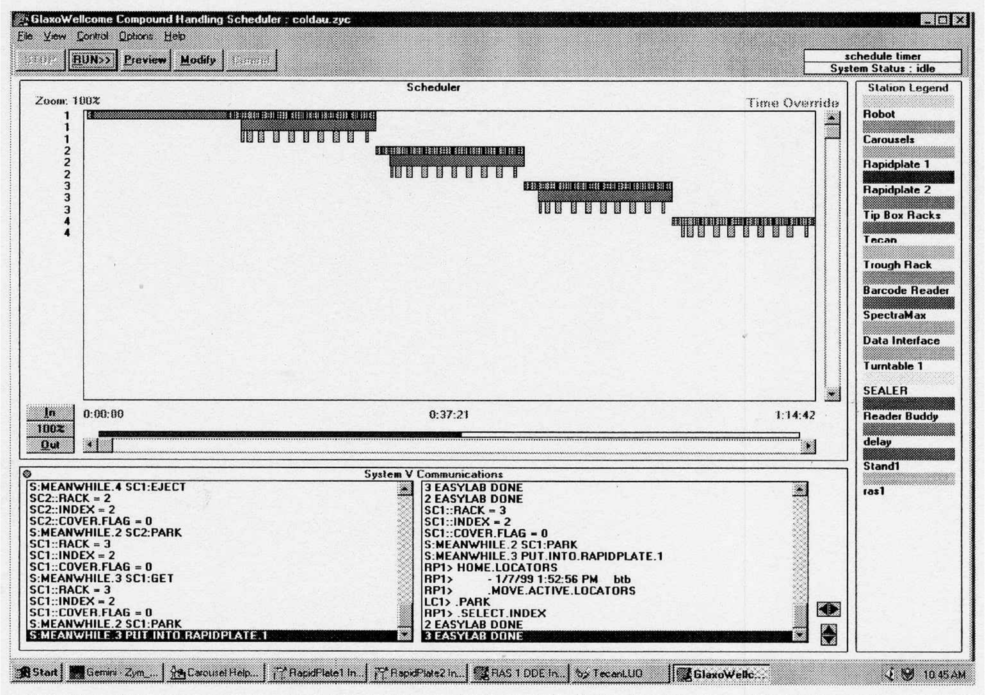

(B)

*Figure 3.* (A) *Gantt chart (Zymark PCS) showing the scheduling of the two simultaneous processes of the production of a serial dilution and the subsequent distribution of the diluted chemistry to assay plates. It can also be observed that the system's efficient interaction with each peripheral maximizes the moves executed with an occupied robotic hand.* (B) *Second Gantt chart demonstrating the process in multiple iterations. The assay plate production rate is 32 plates/h with a system capacity of 352 plates.*

plate in hand can save time. Placement of these two transfer positions in an opposing arrangement allows for simultaneous movement of the active copy under the transfer head and exposure of the finished plate in position for hand access (figure 2). Now the arm can return to the carousel occupied with the finished plate in hand to retrieve the next plate. While the last plate was returned and the next plate acquired, a single transfer is dispensed to the active plate.

The quality of the tip seal was maintained by attaching the tips a single time per master for the eight individual

*Table 1. The system is validated through observing the* $r^2$ *of curves fit to the data points and by comparing the derived* $pIC_{50}$ *produced by the automated system with historic values. The data for each plate are derived from the serial dilution master plate. The* $pIC_{50}$ *determined across all eight copy plates are statistically identical.*

| | Replicate plate no. | | | | | | | | | | | | |
|---|---|---|---|---|---|---|---|---|---|---|---|---|---|
| Dye | 1 | 2 | 3 | 4 | 5 | 6 | 7 | 8 | | | | | |
| Replicate CV % | 3.87 | 3.16 | 2.79 | 3.35 | 2.12 | 2.70 | 2.25 | 2.72 | | | | | |
| µl | 0.92 | 1 | 0.95 | 0.93 | 0.99 | 0.94 | 0.9 | 0.9 | | 0.94 | 0.038 | 3.99% | |
| | $pIC_{50}$ | $pIC_{50}$ | $pIC_{50}$ | $pIC_{50}$ | $pIC_{50}$ | $pIC_{50}$ | $pIC_{50}$ | $pIC_{50}$ | $r^2$ | Average | SD | %CV | Historic |
| Compound 1 | 6.97 | 6.91 | 6.98 | 6.97 | 7.06 | 7.07 | 7.03 | 7.10 | 0.998 | 7.01 | 0.065 | 0.92 | 6.9 |
| 2 | 8.15 | 8.09 | 8.09 | 8.11 | 8.26 | 8.12 | 8.14 | 8.17 | 0.998 | 8.14 | 0.054 | 0.66 | NA |
| 3 | 7.16 | 7.09 | 7.26 | 7.16 | 7.24 | 7.23 | 7.17 | 7.35 | 0.996 | 7.21 | 0.081 | 1.12 | 7.3 |
| 4 | 7.36 | 7.24 | 7.32 | 7.34 | 7.37 | 7.45 | 7.46 | 7.41 | 0.998 | 7.37 | 0.072 | 0.98 | 7.1 |
| 5 | 7.71 | 7.63 | 7.75 | 7.64 | 7.67 | 7.72 | 7.67 | 7.717 | 0.997 | 7.69 | 0.044 | 0.57 | 7.72 |
| 6 | 8.76 | 8.76 | 8.75 | 8.74 | 8.88 | 8.83 | 8.70 | 8.78 | 0.998 | 8.78 | 0.055 | 0.62 | 8.81 |
| 7 | 9.21 | 9.02 | 9.19 | 8.92 | 9.14 | 9.11 | 9.10 | 9.11 | 0.997 | 9.10 | 0.091 | 1.00 | 8.95 |
| 8 | 8.09 | 7.98 | 7.94 | 8.02 | 8.07 | 8.07 | 8.04 | 8.10 | 0.998 | 8.04 | 0.058 | 0.72 | 8.03 |

transfers. The new rapid plate software (96/384 standalone v. 1.01) that allows for more control over pipetting functions provided the control to achieve 3% CVs on 1 µl transfers. This accurate transfer maintains the quality of the original serial dilution and guarantees the assay data to be representative of 'true' values.

### Conclusion

With the high demand for compound selectivity within systems-based research, there is a need to distribute compounds on a regular basis to several targets at once. To turn around data in a timely fasion, it is appropriate to distribute all compounds of a single serial dilution format to a set of multiple replicate assay plates. All the components necessary to handle these sets of selectivity compounds from serial dilution to distribution to assay plates are employed in this single-track system. A TECAN Genesis 150 for the serial dilutions was combined with a Zymark track system's carousels and rapid plate.

Obstacles including an automated standard dilution application, small volumes, variable serial dilutions, speed, carry-over and the future of 384-well formats were all addressed by using disposable polypropylene tips, low-volume syringes and Gemini software for optimal liquid-handling control.

Increasing the efficiency of the machine by traveling between peripherals with an occupied hand is accomplished by using the RapidPlate as a turnstile rather than a fully loaded deck and the staggered carousel racks as a supplier. Locating plates in opposing positions on the RapidPlate turntable allows the dispense head to toggle between the two positions while exposing the opposite plate position to the arm. In this manner, the arm is constantly feeding and replacing the RapidPlate so that while it is dispensing to one daughter, the finished daughter is being sealed and housed and another plate is replacing the vacated position. Copies of a master plate are being made while the next master plate is being serially diluted.

Validity of the entire system that confirms the accuracy of all the liquid-handling processes is evident in the comparison of control data to historic values and goodnes of fit ($r^2$) statistics on new dilutions.

There are several advantages to distributing for secondary screening in this fashion. This programme allows for minimization of compound stores required for distribution to targets. The amount of compound needed to distribute from one serial dilution of a compound to multiple targets is the same amount previously required by each individual scientist. The reduction in the amount of compound distributed lessens the burden on the chemist and compound distribution services. There is timely turnaround of distribution from the time of serial dilution to the time of plating. The number of freeze–thaw cycles is deeply diminished since all assays are plated in one session rather than plating on an as-needed basis. Because a single box of tips is used per mater plate distribution to all copy plates, the cost of consumables is greatly decreased. And perhaps most importantly, this program yields good compromise for bench-to-data between chemists and biologists.

The current capacity of this system with three carousels is 44 masters with eight different assay plate definitions, totaling 352 copy assay plates. The production rate of the system is 32 plates/h, taking 11 h to accomplish the full capacity of the system.

